# The Effect of Far-Red Light on the Growth of Tobacco Leaves

**DOI:** 10.3390/plants14162520

**Published:** 2025-08-13

**Authors:** Lei Liu, Shujie Gai, Chuanke Liu, Zouguo Zeng, Xudong Tan, Jiawei Li, Zhi Zhou

**Affiliations:** 1 College Agronomy, Hunan Agricultural University, Changsha 410128, China; 13762917337@163.com (L.L.); m13249459991@163.com (J.L.); 2 Chemical Materials for Agricultural Cross Disciplinary Joint Laboratory, Hunan Agricultural University, Changsha 410128, China; gaishujie@ahau.edu.cn (S.G.); 15197967263@163.com (C.L.); 14773800916@163.com (Z.Z.); 19158373576@163.com (X.T.); 3 Hunan Provincial Engineering Technology Research Center for Optical Agriculture, Changsha 410128, China

**Keywords:** far-red light, tobacco leaf, cell expansion, shade reaction

## Abstract

To investigate how far-red (FR) light affects tobacco leaf growth, we established different light conditions, namely, CK: white (WL), T1: red (R), T2: red–white (R+WL) combination, T3: white–far-red (WL+FR) combination, and T4: white–red–far-red (WL+R+FR) combination; conducted supplemental light experiments on tobacco; and evaluated the growth of tobacco leaves by determining the biomass, size of the leaves, etc. In addition, the auxin (IAA) content and expression of leaf growth-related genes were examined to further reveal the mechanism of the FR regulation of tobacco leaf growth. The results show a maximum reduction in leaf area size of more than 90% and in fresh dry mass of more than 85%, while the chlorophyll content increased by more than 28%. in tobacco leaves exposed to FR compared with those exposed to white light. Meanwhile, levels of auxin IAA were increased by 113% (T3) and 17% (T4) under far-red light treatment. The anatomical structure of the tobacco leaves showed that FR reduced the number of epidermal cells in the leaves but increased the cell size. Subsequent findings revealed that FR’s impact on leaf growth was mediated through the *PHYB–PIF7–IAA* signaling pathway, wherein it regulated cell division and growth-related genes. This substantiates that FR diminishes the tobacco leaf area by impeding cell division rather than inhibiting cell growth. In this study, we explored the effects of far-red (FR) light on tobacco leaf growth changes and constructed a model of the related signaling pathways. Our results reveal a novel mechanism by which far-red light regulates the growth of tobacco leaves, elucidating how far-red light affects their growth and response to shading conditions. This finding not only provides a scientific basis for the optimization of high-density tobacco planting but also helps to improve photosynthetic efficiency and yield, providing strong support for the sustainable development of tobacco farming.

## 1. Introduction

Tobacco (*Nicotiana attenuata*) is an economic crop primarily harvested for its leaves. The growth and development of tobacco leaves directly affect the yield and quality of the tobacco. In the process of cultivation, improving plant resistance and planting density is a key step in improving tobacco yield. Intensively planted tobacco populations experience reduced air circulation and light transmission, resulting in shade avoidance syndrome (SAS) in the form of reduced leaf area, longer petioles, reduced tolerance, increased amounts of phytovolatiles, and reduced resistance to decline, which, in turn, affects the tobacco yield and quality [1,2,3,4]. The conditions that elicit a shading response are primarily caused by two signaling factors: an increased proportion of FR, and decreased light intensity [5,6]. Leaves serve as the largest site of life activity in a plant body in terms of the proportion of the area exposed to air. The effect of an increase in the proportion of FR on the leaf morphology and structure is strongly correlated with the plant’s ability to photosynthesize [7,8].

Extensive research has shown that increasing FR increases the net photosynthetic rate of plant leaves while decreasing the leaf thickness [7,9,10,11,12]. Studies have shown that with additional FR, rice can achieve greater biomass and tillers because the inclusion of FR enhances the light-absorbing capacity of photosystem I, while shorter wavelengths of light are taken up in photosystem II (e.g., blue light). This is also known as the Emerson effect [8,13,14]. In environments with a low red-to-far-red light ratio (R/FR), plants have the ability to decrease the amount of palisade cell tissue and, consequently, their overall leaf thickness. This can be achieved by reducing the size of chloroplasts and the number of cells within the leaf [15,16,17]. The introduction of far-red light boosts the leaf length and reduces the leaf width in *Arabidopsis thaliana* and *Glycine max*, resulting in plant leaves with an elongated shape compared with the control [18,19]. It was found that shade reduced the number of leaves, total leaf area, and individual leaf area in Arabidopsis and that the total leaf area also depended on the amount of time that the leaf received shade cues during its development. At later stages of development, shade only regulates cell expansion, whereas at earlier stages of development, the inhibitory effect of phytochromes suppresses cell division [20]. Some researchers have posited that, under conditions of low photosynthetically active radiation, cell expansion becomes a more critical factor in leaf growth than cell division. In contrast, other studies indicate that it is the quantity of cells rather than their size that leads to decreased leaf size in plants exposed to low R/FR levels compared with high R/FR levels [7]. It is not known whether the leaf area is ultimately determined by cell division or cell expansion. Recent advances in molecular research have identified many genes that participate in leaf growth and development. These findings offer a rationale for studying the effects of shade on leaf growth.

Recent advances in molecular research have identified many genes that play a role in leaf growth and development. For example, *OsARF2 (auxin response factor 2)*, an uORF-free inhibitory ARF, may synergize with other *ARFs* to regulate leaf development by balancing auxin signaling [21]. *EBP1 (ErbB3-binding protein 1)*, an auxin-regulated ribonucleoprotein, orchestrates cell proliferation and expansion by integrating ribosomal biosynthesis with cell cycle progression in a dose-dependent manner, ultimately determining the plant organ size [22]. *ANT (AINTEGUMENTA)* genes are regulated by outer bead pericycle cell proliferation and auxin synthesis, which directly affect the development and size of plant organs and other organs [23]. The *CYCD3 (CYCLIN D3)* gene, which encodes a D-type cyclin involved in the plant cell cycle, is pivotal in the development of shoot lateral organs. It orchestrates cell proliferation, impacts cell number determination, and modulates endoreduplication and cell size. By integrating these critical developmental processes, *CYCD3* profoundly shapes organ growth patterns. In the context of leaf development, *CYCD3* operates downstream of *ARGOS (Auxin-Regulated Gene involved in Organ Size)* and *ANT* and is positioned downstream of AMP1 and cytokinins within the shoot apical meristem (SAM). This strategic placement underscores the gene family’s essential role in harmonizing cell proliferation with overall development, particularly in the context of hormonal signaling pathways [24]. These findings provide a theoretical basis for the study of the effects of shade on leaf growth. *PHYB (photosensitive pigment B)* is a red and far-red light receptor that regulates plant growth and development by sensing light changes. In shaded environments, a lower R/FR ratio results in a shift to an inactive state of *PHYB*. As the number of inactive forms of *PHYB* increases, its overall activity decreases, which, in turn, suppresses the activity of *PIFs (phytochrome-interacting factors)*. As a result, *PIFs* act as transcription factors that promote growth-associated gene expressions and facilitate plant growth [25,26]. Studies have shown that *AtAN3 (ANGUSTIFOLIA3)* is a key factor in promoting leaf cell proliferation through photosensitizing pigments and that *AtPIF7 (Phytochrome-interacting factor 7)* is a potent inhibitor. FR releases *AtPIF7* by inactivating *AtPHYB*, and *AtPIF7* interacts with *AtAN3*, which ultimately reduces leaf size. Exposure to FR in Arabidopsis activates *AtPIF7*, which then displaces and substitutes *AtAN3* at the targeted promoter site, leading to the inhibition of *AtAN3* activity. This interaction results in opposing regulatory effects on a collection of genes, indicating a cis-acting promoter component shared between *AtAN3* and *AtPIF7*. Thus, variation in the outside light environment could be dynamically managed to manipulate the cellular expression of growth-related genes and to adjust plant leaf growth [27]. Upon exposure to far-red light, the *PHYB* photoreceptor undergoes a conformational change, leading to the activation of transcription factor *PIF7*. *PIF7* plays a crucial role in regulating the expression of genes involved in cell elongation and photosynthesis. Specifically, *PIF7* promotes the synthesis and transport of the plant hormone auxin (IAA), which is essential for cell elongation and overall plant growth [28]. It was found that shade could induce the activation of a range of genes related to cell proliferation and expansion in soybean and that this induction was influenced by the leaf developmental stage. Moreover, shade exposure leads to a substantial increase in the levels of auxin and gibberellins, along with a significant decrease in cytokinin content in young, mid-aged, and old leaves, indicating the potential crucial roles of auxin, gibberellin, and cytokinin in this regulatory process [29]. Moreover, it has also been shown that under shade conditions, *NtZTL (ZEITLUPE is one of the core circadian rhythm regulatory genes in Nicotiana attenuate)* in tobacco directly interacts with *NtPHYB* to govern the transcript levels of *NtPHYB*, *NtPIF3a*, *NtPIF7*, and *NtPIF8*. However, the precise mechanism through which *NtZTL* modulates *NtPIF* remains unclear. *NtZTL* affects the expressions of leaf stunting and the shade marker gene *ATHB2*, which may be related to the regulation of auxin and *NaPHYB1*-related signaling [30].

In this study, we investigated the effects of FR on tobacco leaf growth by combining physiological, anatomical, and molecular analyses. We hypothesized that FR decreases leaf area by impeding cell proliferation through the *PHYB–PIF7–IAA* pathway rather than inhibiting cell expansion. Our findings aimed to elucidate the regulatory network underlying shade adaptation in tobacco, providing a theoretical foundation for improving yield in dense planting systems.

## 2. Materials and Methods

### 2.1. Plant Material and Treatment

The experimental site was located in the plant growth room of the Optical Agricultural Engineering Research Center, Hunan Agricultural University, Hunan Province, China (27.55° N, 113.92° E), with a temperature of 24 ± 2 °C and humidity of 80 ± 10%. Tobacco seedlings were grown on culture racks with dimensions of L (200 cm) × W (60 cm) × H (220 cm), which were uniformly divided into four layers according to height. The culture racks were fitted with LED lights, which were adjusted in height according to the tobacco seedling growth to maintain uniform and consistent illumination. In addition, each culture rack was equipped with a black shade cloth to isolate the interference of external light.

**Figure 1:**
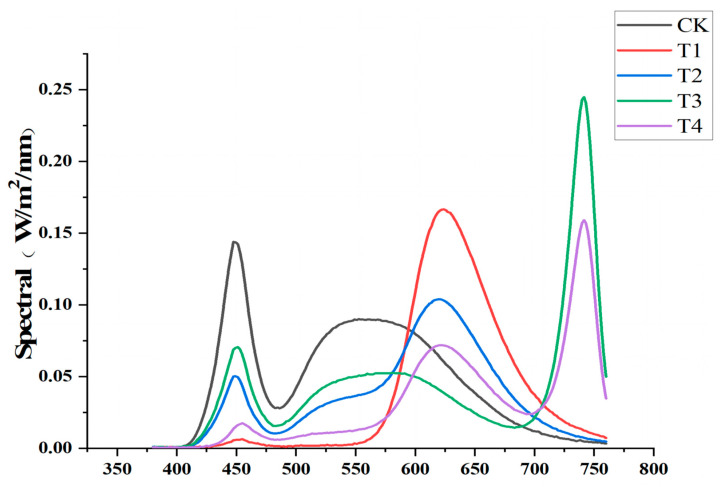
Spectrogram of the LED: WL, R, WL+R, WL+FR, and WL+R+FR. Measurements were obtained using the SPIC-200 spectral chromaticity illuminance meter.

The *Xiangyan No.7* and *Yunyan87* varieties were selected for this study (provided by Changsha Tobacco Company); both varieties are widely grown in southern China. The seeds were sown in seedling trays that were 50 cm long, 30 cm wide, and 5 cm high (in which the substrate consisted of vermiculite, peat, and straw fermentation material). These were placed under full-spectrum white light for germination. The LED light quality used is shown in Figure 1 as CK (WL). The light lamp was provided by Changsha Spot Lighting Co. (Changsha, China). When the seedlings reached the seedling emergence stage (approximately 10 days after sowing), they were transplanted into plastic pots containing the same substrate, with a top area of 10 × 10 cm, a bottom area of 5 × 5 cm, and a height of 8 cm, and then subjected to different LED light treatments, which were CK (WL), T1 (R), T2 (WL+R), T3 (WL+FR), and T4 (WL+R+FR); the LED spectra used are shown in Figure 1. Each variety was planted in 12 pots per treatment; the light intensity was maintained at 105 ± 20 μmol m^−2^s^−1^, and the light duration was 16 h/day. The R/FR ratio was obtained by calculating the integral areas in the 570–690 nm (red light) and 690–760 nm (far-red light) ranges. The R/FR ratio for each treatment was 12, 24, 8, 0.5, and 1, respectively (Table S1). All spectra and light intensities were measured using a SPIC-200 spectral color illuminometer (Yuanfang Optoelectronics, Hangzhou, China). When plants reached the rooting stage (45 days), five plants from each treatment of each variety were randomly selected, tested, and analyzed. Note: Although we selected 5 plants per treatment group, we generally only used 3 plants for the experiment, with the remaining 2 serving as backup material in case of insufficient experimental material or damage to the experimental material.

### 2.2. Determination of Leaf Morphological Parameters and Biomass

We selected 3 uniformly growing tobacco plants from each variety within each treatment group. Subsequently, the third, fourth, and fifth leaves of each treated tobacco plant were picked for accurate measurements of the leaf area, length, and width. Digital images of the leaf blades were captured using a Leaf Analyzer (Wanshen Inspection Technology Co., Ltd., Guizhou, China), and these parameters were quantified using the Wanshen LA-S Leaf Area Analysis System (Wanshen Inspection Technology Co., Ltd., Guizhou, China). Following a 45-day growth period, the fresh leaf mass from five plants of each tobacco variety was initially assessed using an electronic balance (Ohaus AX224Z, Shanghai, China). These samples were then heated for 30 min at 105 °C and dried at 80 °C until the tobacco reached a constant mass. Subsequently, the mass of the dried leaves, i.e., the dry mass, was measured using the same electronic balance (Ohaus AX224Z, Shanghai, China) as described above.

### 2.3. Anatomy of the Leaf Blade

To observe leaf epidermal structure, we first randomly selected 3 uniformly growing tobacco plants from each variety within each treatment group. We then washed the fourth leaf of each plant and dried it with absorbent paper. Colorless nail polish was then applied evenly on both sides of the leaf blade. Once the colorless nail polish dried, a film formed on the surface of the leaf blade. This film was gently removed with tweezers and cut into a piece measuring approximately 1 × 1 cm, which was imprinted with the structure of the stomata in the epidermis. The film was placed on a slide with a drop of distilled water, and a coverslip was slowly placed along the side of the water drop to prevent air bubbles from forming, creating a temporary stomatal slide. This slide was then observed using a light microscope.

Stomatal conductance was measured using the LI-6800 portable photosynthesis measurement system (LI-COR, Lincoln, NE, USA). During the measurement process, the leaf chamber of the photosynthesis meter was fixed in the middle of the tobacco leaf to ensure that the leaf was flat and well-sealed with the leaf chamber. The photosynthesis meter automatically calculates stomatal conductance using a formula based on Fick’s diffusion law, which derives stomatal conductance from the vapor pressure difference between the inside and outside of the leaf and gas flow rate. The experiment was conducted under natural light conditions, with measurements taken between 9:00 a.m. and 11:00 a.m. to avoid the effects of light saturation and light inhibition on the results. Three tobacco plants were selected for each treatment group, with 3 functional leaves measured per plant, for a total of 9 replicates.

Three tobacco plants were selected for each treatment group. Leaf slice measurements were performed on the fourth leaf of each tobacco seedling. Using a razor blade, a 1.5 × 1.5 cm section was extracted from the mesophyll near the main vein. The slices were then quickly fixed and preserved in a standard fixative solution, which consisted of 5 mL of formaldehyde (37–40%), 5 mL of glacial acetic acid, 90 mL of 50% alcohol, and 5 mL of glycerol per 100 mL of fixative. Initially, the material was dehydrated through a series of ethanol solutions with increasing concentrations—70%, 80%, 90%, 95%, and 100%—for 20 min each using a JJ-12J dehydrator (Wuhan Junjie Electronic Co., Ltd., Wuhan, China). After dehydration with 100% ethanol (fresh) for 20 min, a paraffin mold (metallic) was slightly heated on an alcohol lamp using tweezers. It was then placed on a flat tabletop, and a wax cup filled with pure paraffin wax removed from the incubator. The pure paraffin wax was poured into the wax cup. Forceps were heated slightly on the alcohol lamp, and the material was clipped, placed cut side down into the wax mold, and arranged neatly. The molten wax was then gently poured into the embedding box. The embedded samples were sliced using a paraffin slicer (Shinki BK-2258, BIOBASE, Jinan, China) to prepare paraffin sections for observation under an optical microscope. The process began with a series of solutions using turpentine and alcohol, which included sequential treatments with 50%, 70%, 95%, and 100% alcohol; a mixture of 50% pure alcohol and 50% turpentine; a solution of 1/3 alcohol and 2/3 pure turpentine; and finally, two stages of turpentine (1) and (2). The sections were immersed for 10 s in each solution before pure alcohol was used, and then for 1, 2, 3, and 5 min in the subsequent stages. The tissue sections were dewaxed and rehydrated following this procedure. Toluidine blue staining was applied to enhance the visibility of the sections. They were then immersed in graded alcohols of 50%, 70%, and 80% for 3 to 8 s each. Finally, the sections were clarified with xylene for 5 min and sealed with a neutral adhesive. This meticulous process ensured that the paraffin sections were of high quality and suitable for viewing under a light microscope (ML31, Mingmei, Guangzhou, China). The data obtained from these observations were quantified using ImageJ1.53 (National Institutes of Health, Bethesda, MD, USA).

### 2.4. Photosynthetic Characterization

Chlorophyll fluorescence parameters: 3 tobacco plants were selected for each treatment group. Measurements were performed with a FluorPen handheld chlorophyll fluorometer (FluorPen110, Dianjiang, Shanghai, China). This involved fully acclimatizing the leaves to light prior to the measurement, followed by a 10 min darkness treatment. Each tobacco plant was measured three times, using the 3rd, 4th, and 5th leaves, respectively [31]. The OJIP test was performed first. The fluorescence induction curves from the O (initial fluorescence), J, I, and P (maximum fluorescence) stages were recorded. The dark treatment was repeated to measure the variable fluorescence (Fv), photochemical burst coefficient (QP), and maximum photosynthetic efficiency (R) of PSII, and each measurement was repeated three times and averaged.

 Fv=FM−F0;

$$\mathrm{qP}\;=\left(\mathrm{FM}-\mathrm{Fs}\right)/\;\mathrm{Fv};$$

R=Fv/FM.

FM—maximum fluorescence intensity;
Fs—steady-state fluorescence intensity;
F0—initial fluorescence intensity.

Photosynthetic parameters: First, 3 uniformly growing tobacco plants of each variety were randomly selected from each treatment group. Then, the third tobacco leaf of each plant was chopped (removing the thick veins) and 0.100 g of fresh leaves were weighed. The weighed fresh leaves were added to a 10 mL centrifuge tube, followed by 10 mL of an acetone–ethanol mixture (80% acetone and 95% ethanol by volume, 1:1), and extracted in the dark for 8 h. The supernatant was then centrifuged at 12,000 rpm for 1 min to determine the absorbance values. The absorption of the supernatant was determined using an enzyme marker (Infinite^®^ 200 PRO, Tecan, Singapore) at room temperature at 645 nm and 633 nm using the corresponding reagent (acetone–ethanol mixture) as a reference. The formulas for calculating the chlorophyll content are listed below [32]:

$$\;\mathrm{Chl}\;a\;\left(\mathrm{mg}/g\right)\;=\;\left(12.72\;\mathrm{OD}663-2.59\mathrm{OD}645\right)\;V/1000W$$

$$\mathrm{Chl}\;b\left(\mathrm{mg}/g\right)=\left(22.88\;\mathrm{OD}645-4.67\mathrm{OD}663\right)\;V/1000\;W$$

$$\mathrm{Chlorophyll}\;\mathrm{content}\;\left(\mathrm{mg}/g\right)=\;\mathrm{Chl}\;a+\mathrm{Chl}\;b$$

Chl a—chlorophyll a;
Chl  b −chlorophyll b;
V—volume of extract (mL);
W—mass (g);

The conversion factor is 1000.

### 2.5. Extraction of the Total RNA and Analysis Using Real-Time qPCR

Real-time quantitative reverse transcription polymerase chain reaction (RT-qPCR) was conducted using information on the tobacco homologous genes and primers supplied with the Supplementary Materials (Table S2). qPCR was performed using 3 uniformly growing tobacco plants of each variety that were randomly selected from each treatment group. Then, the 5th leaf of each plant was measured. The total RNA was isolated and purified using the same method as Zhou et al. [33]. Next, a nanophotometer manufactured by IMPLEN, Inc. was utilized to measure the concentration of the RNA samples. The cDNA was synthesized using reverse transcription from the total RNA template according to the guidelines for the use of All-in-One RT SuperMix provided by Vazyme. A 10 µL reaction system was used to determine the relative expression of specific genes in the tobacco using the RT-qPCR technique. The data were analyzed according to the user guide of the BIORAD CFX96 Touch real-time quantitative PCR instrument. When the fluorescence intensity of a reaction exceeded a set threshold, the 2^−ΔΔCt^ method (where Ct represents the number of threshold cycles) was used to calculate the relative expression of the sample [34]. The CK treatment was used as a control sample, i.e., the sample in relation to which a change in the expression of the tested genes was detected. Three independent measurements were performed for each sample in the experiment to ensure the accuracy of the results. *NtActin* was utilized as an internal reference gene. Using this method, we monitored the expression of the following genes: *ARF2 (auxin response factor 2)*, *EBP1 (ErbB3-binding protein 1)*, *ANT (AINTEGUMENTA)*, *CYCD3 (CYCLIN D3)*, *PHYB (photosensitive pigment B)*, and *PIF7 (Phytochrome-interacting factor 7).*

### 2.6. Hormone Analysis

First, three uniformly growing tobacco plants of each variety were randomly selected from each treatment group. Subsequently, 0.2 g of leaf tissue was cut from the fifth leaf of each plant and placed in a volumetric flask containing 10 times the volume of acetonitrile. The sample was incubated with acetonitrile at 4 °C overnight. Afterwards, the sample was centrifuged at 12,000 rpm for 5 min to collect the centrifuged supernatant. Next, 5× the acetonitrile volume was added to the collected supernatant for precipitation, and finally, the supernatant was collected again. The two supernatants were mixed. Then, 50 mg of C18 packing was added to the supernatant, followed by 30 s of vigorous shaking. The supernatant was then centrifuged at 10,000 rpm for 5 min, removed, and blown dry using nitrogen. Afterwards, the dried sample was redissolved with 200 µL of methanol and filtered through a 0.22 µm organic phase filter membrane. Finally, the filtered samples were stored at −20 °C for subsequent assays. The phytohormone standard sample, IAA, was purchased from Sigma-Aldrich. IAA was dissolved in 2 mL of methanol solution containing 0.1% formic acid to prepare a standard solution with a final concentration of 500 µg/mL and then kept at −20 °C as a storage solution. The 2 μL storage solution (500 μg/mL) was diluted to 990 μL of methanol. We obtained a final concentration of 1 μg/mL as the primary solution. Different primary solution volumes were aspirated into test tubes (1.5 mL tubes). The volume in the test tube was adjusted to 1 mL final concentrations of 0.1 ng/μL, 0.2 ng/μL, 1 ng/μL, 5.0 ng/μL, 20.0 ng/μL, 50.0 ng/μL, and 200.0 ng/μL using an appropriate amount of methanol. For the organic phase, 900 mL of chromatographically pure methanol and 1 mL of formic acid were added sequentially to a volumetric flask. Methanol was added to the volumetric flask until the volume reached 1 L, after which the bottle was inverted for thorough mixing. For the inorganic phase preparation, 900 mL of deionized water was first added to the volumetric flask, followed by 1 mL of formic acid. The volume in the volumetric flask was adjusted to 1 L with deionized water and mixed by inversion. The analysis of plant hormones was accomplished using HPLC-MS/MS techniques performed on a 1290 HPLC system (supplied by Agilent, Santa Clara, CA, USA) and a SCIEX-6500 Qtrap mass spectrometer (supplied by AB Sciex, Foster City, CA, USA). During the HPLC analysis, a reversed-phase pore–shell 120 SB-C18 column, manufactured by Agilent in Palo Alto, CA, was used.

### 2.7. Statistical Analysis

Experimental data were used to generate images using Origin 2022 (Microcal Software Inc., Northampton, MA, USA). Subsequently, an analysis of variance (ANOVA) using SPSS 26 was conducted, followed by Duncan’s multiple range test for ANOVA (*p* < 0.05). Following the statistical analyses, the results were recorded using WPS Office.

## 3. Results

### 3.1. Changes in Leaf Morphology of Tobacco Seedlings Under Different Light Conditions

**Figure 2:**
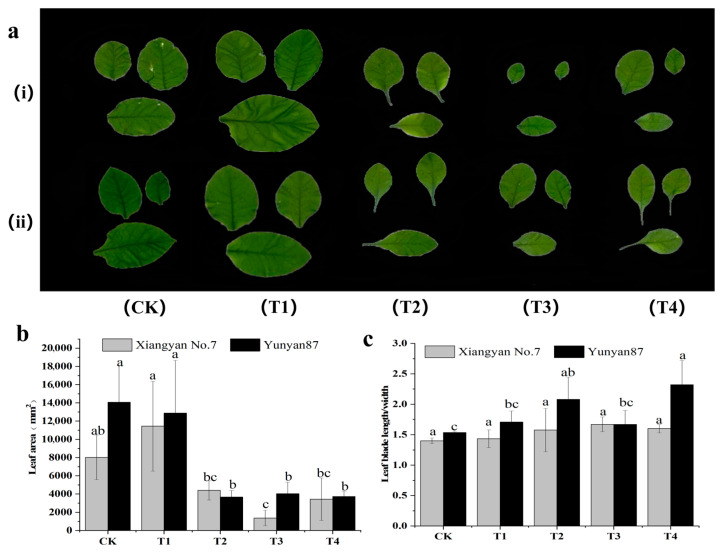
Differences in the leaf morphologies of two tobacco species under different light conditions. (**a**) Photographs of the 3rd, 4th, and 5th leaf blades of representative (i) *Xiangyan No.7* and (ii) *Yunyan87* seedlings grown under different treatments. (**b**) The effect of varying light conditions on the leaf area of *Xiangyan No.7* and *Yunyan87* tobaccos. (**c**) The leaf blade aspect ratios of the *Xiangyan No.7* and *Yunyan87* tobaccos under different light conditions, where different letters indicate that there was significant variation between the treatments (*p* < 0.05).

Tobacco growth and development directly affect tobacco yield. To study the differences in tobacco growth and development under different spectra, we compared the leaf areas of the control and experimental groups. The results show that the *Xiangyan No.7* tobacco leaf area increased by 42.7% (T1) but decreased by 35.3% (T2) under the red light treatment. It was reduced by 90.7% (T3) and 73.4% (T4) under the far-red light treatments. The *Yunyun87* tobacco showed 5.4% (T1) and 60% (T2) reductions in leaf area under the red light treatment, and reductions of 71.3% (T3) and 73.4% (T4) under the far-red light treatment (Figure 2a,b). The leaf LW ratio of Yunyan87 tobacco seedlings was significantly higher under T2 and T4 treatments (Figure 2c). Through the above experimental results, we found that tobacco plants grew more slowly under far-red (FR) light and developed narrower, longer leaves.

### 3.2. Changes in Tobacco Seedling Biomass Under Different Light Conditions

**Figure 3:**
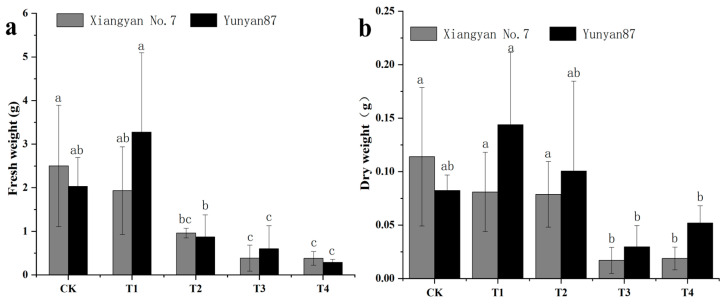
Analysis results of differences in fresh (**a**) and dry masses (**b**) of tobacco seedlings under different light conditions. Different letters indicate that there was a significant variation between the treatments (*p* < 0.05).

Biomass is a crucial indicator for assessing the growth status of plants. To investigate how various light qualities impact tobacco seedling biomass, we measured the dry mass of aboveground parts of tobacco. The results showed reductions in the fresh tobacco mass by up to 85.7% and in the dry mass by up to 85.2% under far-red light treatment. Specifically, compared with CK, the reduction in fresh seedling mass of *Xiangyan No.7* was significant under T2, T3, and T4. The fresh weight of *Yunyan 87* decreased significantly under both T3 and T4 conditions (Figure 3a). For *Yunyan 87*, no statistically significant differences in dry matter weight were observed between CK and the other treatments, whereas for *Xiangyan 7*, a significant reduction occurred under T3 and T4 (Figure 3b). The fresh and dry mass of the tobacco seedlings under the four treatments showed that the FR treatments had relatively decreased the fresh and dry mass.

### 3.3. Changes in Tobacco Seedling Photosynthetic Characteristics Under Different Light Conditions

**Figure 4:**
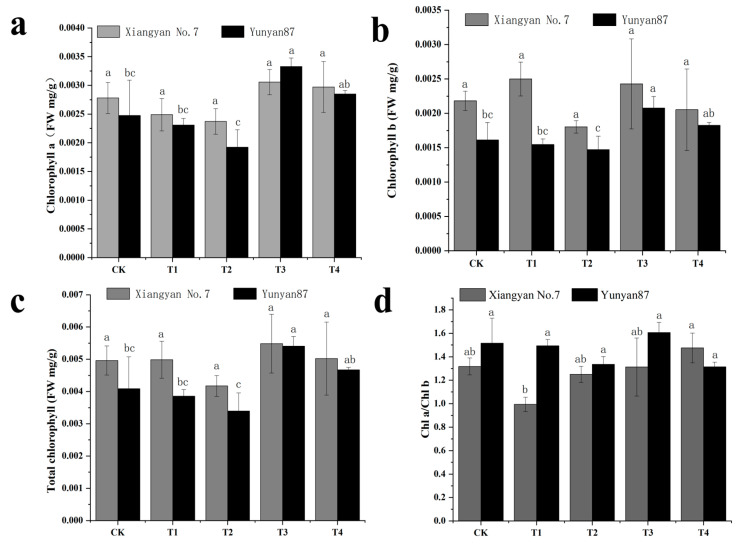
(**a**) Chlorophyll a content; (**b**) chlorophyll b content; (**c**) difference in total chlorophyll content; and (**d**) the ratio of chlorophyll a to chlorophyll b of *Xiangyan No.7* and *Yunyan87* under different spectral irradiations; different letters denote significant differences between the treatments (*p* < 0.05).

The results of the study on the effects of different light conditions on the growth of *Xiangyan No.7* and *Yunyan 87* showed that, under far-red light treatment, chlorophyll a (a) in tobacco leaves increased by 27.7%, chlorophyll b (b) increased by 29.2%, and total chlorophyll increased by 28.7%. Specifically, *Yunyan 87* showed a significant increase under the T3 treatment (Figure 4a). Compared with CK, there were no significant differences in chlorophyll b content among the different light conditions for *Xiangyan No.7* (Figure 4b), while *Yunyan 87* showed a significant increase under T3 treatment (Figure 4b). Compared with CK, there were no significant changes in total chlorophyll content among the four treatment conditions for *Xiangyan No.7* (Figure 4c); *Yunyan 87* showed a significant increase under T3 treatment (Figure 4c). Compared with CK, the chlorophyll a/b ratio of *Yunyan 87* and *Xiangyan No.7* showed no significant changes under any treatment (Figure 4d). In summary, far-red light can increase chlorophyll content in tobacco leaves.

**Figure 5:**
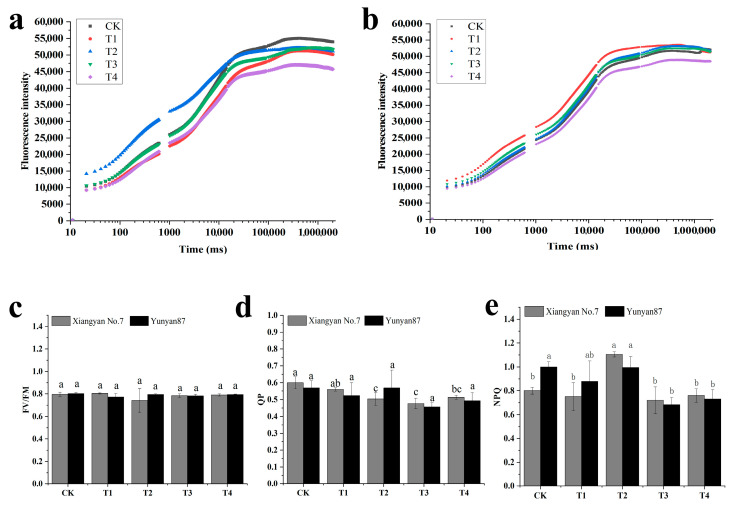
(**a**) OJIP curve of *Xiangyan No.7* under different light quality conditions; (**b**) OJIP curve of *Yunyan87* under different light quality conditions; (**c**) FV/FM; (**d**) NPQ; (**e**) QP. Different letters indicate that the treatments were significantly different from each other (*p* < 0.05).

“OJIP” refers to the fast chlorophyll fluorescence induction curve, which starts from point “O”, the lowest or starting point of fluorescence. Points “J” and “I” mark the transition of the curve from the starting point to the highest point “P”, and the fluorescence intensity at point “P” reflects the maximum fluorescence of PSII under light-saturated conditions. The peak height of the “OJIP” curve is an indicator for evaluating the light energy capture efficiency in plants, and the higher the value, the stronger the plant’s ability to utilize light energy. The OJIP curves changed significantly after different spectral irradiation treatments, but the effects of different light quality treatments on the two species’ OJIP curves were not consistent. Compared with CK, the OJIP peaks of the *Xiangyan No.7* seedling leaves were higher than those under T1, T2, and T3, whereas the peaks of leaves under T1, T2, and T3 were comparable. The T4 leaves had the lowest peaks. In *Yunyan 87*, the peaks of the tobacco seedling leaves were comparable under T1, T2, and T3, while the lowest peaks were observed under T4 (Figure 5a,b). Fv/Fm is the maximum photosynthetic efficiency of PSII, which reflects the maximum light energy conversion efficiency of PSII. For the two varieties, Fv/Fm was not significantly changed under the five different spectral irradiations (Figure 5c). Compared with CK, the QP of *Xiangyan No.7* significantly decreased under T2, T3, and T4 (Figure 5d). NPQ represents the non-photochemical quenching coefficient, which indicates the plant’s ability to protect itself from photodamage. In *Xiangyan No.7*, it increased significantly under T2. For *Yunyan87*, NPQ decreased significantly under T3 and T4 (Figure 5e). The higher the NPQ, the more light energy is converted into heat, which is unfavorable for photosynthesis. Fv/Fm reflects the potential of the photosynthetic system, where a high Fv/Fm value may indicate a higher potential of the photosynthetic system. The QP is a measure of the efficiency of the photosynthetic system II (PSII), which reflects the efficiency of the light energy absorbed by the chlorophyll molecules and used for photosynthesis, where a lower QP may indicate higher photosynthetic capacity. In addition, we found that the Plabs (photosynthetic performance index) of both tobacco cultivars decreased significantly under T2 treatment but increased markedly under T3 and T4 treatments. This indicates that far-red light can optimize photosynthetic efficiency, enhance light-harvesting capacity and electron transport efficiency, and promote carbon assimilation (Figures S1 and S2). The QPs of T2, T3, and T4 of *Xiangyan No.7* were significantly lower than that of CK, and the NPQs of the two kinds of tobacco plants were lower under T3 and T4. Therefore, the two tobacco plant species had the best photosynthetic characteristics under T3 and T4.

### 3.4. Anatomical Analysis of Tobacco Seedlings Under Different Light Conditions

**Figure 6:**
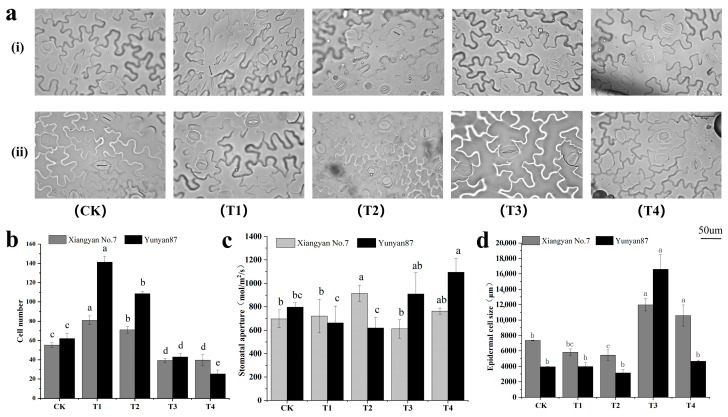
Differences in the tobacco leaf surface cells and stomata under different spectral irradiations. (**a**) Photographs of epidermal cells of tobacco seedlings of *Xiangyan No.7* (i) and *Yunyan87* (ii) under different spectral treatments; (**b**) the cell number of epidermal cells in tobacco leaves exposed to various spectral treatments; (**c**) the stomatal conductance of leaf peridermal cells under different light spectrum processing; (**d**) the size of tobacco leaf epidermal cells under various spectral treatments. Different letters indicate that there was a significant variation between the treatments (*p* < 0.05).

The numbers of epidermal cells in the *Xiangyan No.7* and *Yunyan87* seedlings increased significantly under the T1 and T2 treatments, while decreasing significantly under the T3 and T4 treatments (Figure 6b). The stomatal conductances of the *Xiangyan No.7* seedlings increased significantly under T2 (Figure 6c). The stomatal conductance of the *Yunyan87* seedlings increased significantly under T4 (Figure 6c). The epidermal cell size of the *Xiangyan No.7* seedlings was significantly reduced under T2, and significantly increased under T3 and T4. the epidermal cell size increased significantly under T3 in the seedlings of *Yunyan87 *(Figure 6d).

**Figure 7:**
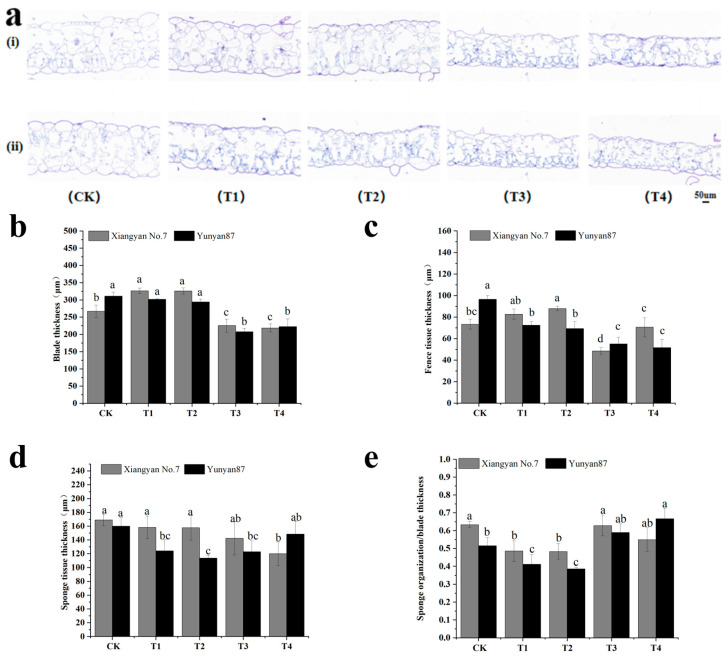
(**a**) Leaf cross-section images of *Xiangyan No.7* (i) and *Yunyan87* (ii) seedlings under different spectra. (**b**) Leaf thickness of tobacco seedlings under different light conditions. (**c**) Barred tissue thickness. (**d**) Spongy tissue thickness. (**e**) Quantitative analysis results of the spongy tissue thickness/leaf thickness. Different letters indicate significant differences (*p* < 0.05) between the treatments.

The chloroplasts in leaves differentiate into fenestrated and spongy tissues to reduce the difference in light exposure between the front and back of the leaf. As shown in Figure 7, the response of the mesophyll tissue to the light environment of different tobacco varieties was not completely consistent: the leaf thickness of the *Xiangyan No.7* seedlings increased markedly under T1 and T2 and decreased significantly under T3 and T4. The leaf thickness of the *Yunyan87* tobacco seedlings decreased significantly under T3 and T4. The results show that red light could encourage tobacco leaf thickening, while FR reduced the tobacco leaf thickness (Figure 7b). The fenestrated tissue thickness of *Xiangyan No.7* increased significantly under T2, while it decreased significantly under T3. For *Yunyan87*, the fenestrated tissue thickness was significantly reduced in all four treatments, with a more pronounced reduction in T3. These findings indicate that red light enhanced the spongy tissue development in the tobacco leaves, while FR reduced the spongy tissue development (Figure 7c). The thickness of spongy tissue in the *Xiangyan No.7* tobacco seedlings decreased significantly under T4. The palisade cell tissues of the *Yunyan 87* tobacco seedlings were significantly reduced under T1, T2, and T3. The growth of spongy tissue in the tobacco leaves was inhibited by different light treatments relative to CK, with the inhibitory effect of FR (T3, T4) being more pronounced (Figure 7d).

The spongy tissue/leaf blade thickness responded to the degree of looseness in the organizational structure of the plant’s chloroplastic tissues. The performance of the spongy tissue thickness/leaf thickness in different light environments was not identical between *Xiangyan No.7* and *Yunyan87*. Under T1 and T2, leaf tissue laxity of the *Xiangyan No.7* seedlings was significantly reduced. The structural relaxation of the leaf flesh organization of the *Yunyan87* seedlings was obviously reduced under T1 and T2, while obviously increased under T4. Therefore, we hypothesized that the organizational structural looseness degrees of tobacco leaf flesh tissue decreased in the red light spectrum and increased in the FR spectrum (Figure 7e).

### 3.5. Gene Expression Analysis Related to Cell Proliferation and Expansion

**Figure 8:**
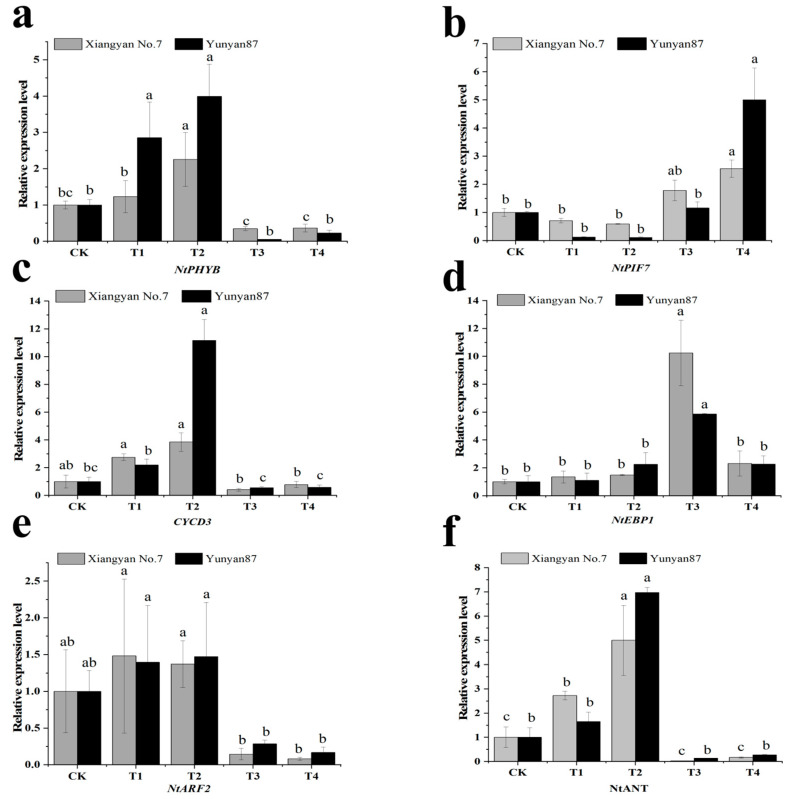
Expression levels of six cell proliferation/enlargement genes under different light treatments. The expression levels were normalized with respect to the internal reference gene *NtActin* and are shown relative to the unity value of CK. (**a**) *NtPHYB*, (**b**) *NtPIF7*, (**c**) *NtCYCD3*, (**d**) *NtEBP1*, (**e**) *NtARF2*, and (**f**) *NtANT*. Different letters on the error bars indicate a significant difference between the treatments (*p* < 0.05).

To investigate the impact of FR on the tobacco cell growth mechanisms, we performed an expression analysis of relevant genes, including the red/FR receptor *NtPHYB* and its downstream transcription factor *NtPIF7*, as well as genes related to cell expansion, namely, *NtARF2*, *NtEBP1*, *NtCYCD3*, and *NtANT*. The results showed that the expression of *NtPHYB*, *NtCYCD3*, *NtARF2*, and *NtANT* in tobacco seedlings was upregulated under T1 and T2 conditions, but downregulated under T3 and T4 conditions. However, not all treatments showed statistically significant differences. Specifically, under T1 treatment, the *NtANT* gene in the *Xiangyan No.7* variety showed a significant increase, while the other three genes showed no significant differences. In the *Yunyan87* variety, the *NtPHYB* gene showed a significant increase, while the other three genes showed no significant differences. Under T2 treatment, the *NtPHYB* and *NtANT* genes in the *Xiangyan No.7* variety showed significant increases, while the other two genes showed no significant differences. And the *NtPHYB*, *NtCYCD3*, and *NtANT* genes of the *Yunyan87* variety showed a significant increase, while the *NtARF2* gene showed no significant difference. In the T3 treatment group, the expression levels of all four genes in both *Xiangyan 7* and *Yunyan 87* showed no statistically significant differences from those of CK. Under T4 treatment, there was no significant difference in the expression levels of four genes in the *Xiangyan No.7* variety. Under T4 treatment, there were no significant differences in the expression levels of four genes in the *Yunyan87* variety (Figure 8a,c,e,f). The *NtPHYB* gene expression in *Xiangyan No.7* increased 2.32-fold under T2 compared with CK, and the *NtPHYB* gene expression in *Yunyan87* increased 2.85-fold under T1 and 3.99-fold under T2 compared with CK. The *NtCYCD3* gene expression of *Yunyan87* increased 10.99-fold under T2 compared with CK. The *NtANT* gene expression of *Xiangyan No.7* increased 2.71-fold under T1 and 5-fold under T2 compared with CK. The *NtANT* gene expression in *Yunyan87* increased 6.97-fold under T2 compared with CK (Figure 8f). The results showed that the expression of *NtPIF7* in tobacco seedlings was downregulated under T1 and T2 conditions and upregulated under T3 and T4 conditions, but not all treatments showed statistically significant differences. Specifically, there were no obvious differences in the expression of the *NtPIF7* gene between the two varieties under T1, T2, and T3 treatments, while the expression of the *NtPIF7* gene in both varieties significantly increased under T4 treatment (Figure 8b). The *NtEBP1* gene expression of *Xiangyan No.7* increased 10.24-fold under T3 compared with CK. The *NtEBP1* gene expression of *Yunyan87* increased 5.26-fold under T3 compared with CK. The expression of the *NtEBP1* gene was upregulated in both tobacco seedlings under T3 treatment conditions (Figure 8d).

### 3.6. IAA Changes in Tobacco Seedlings Under Different Light Conditions

**Figure 9:**
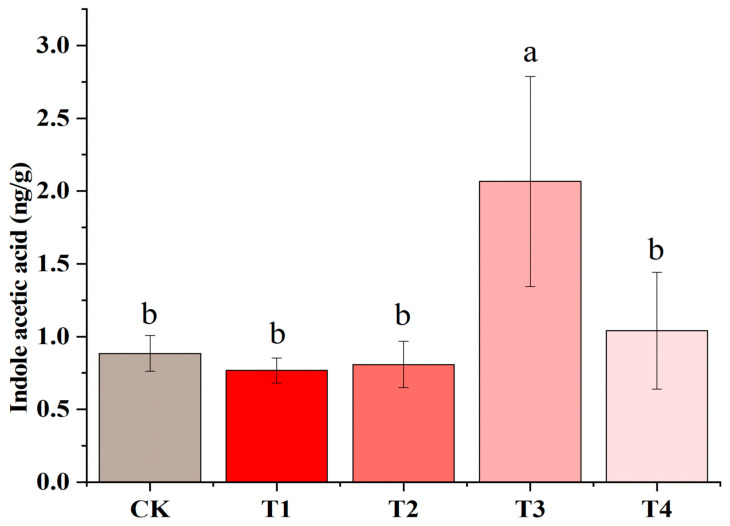
Effects of different light treatments on auxin (IAA) in *Yunyan87* leaves. Different letters indicate that there was a significant variation between the treatments (*p* < 0.05).

The IAA content was measured in the fourth leaf from the bottom of the tobacco seedlings. The results indicate that the tobacco IAA increased up to 133.6% under the far-red light treatment. Specifically, compared with CK, the auxin IAA content was significantly higher under T3 than under CK (Figure 9), indicating a potential promotion of auxin IAA synthesis by FR. However, the FR-rich T4 could not induce a significant increase in auxin, perhaps because of the difference in the ratio of red light to FR, which may have affected the distribution and activity of auxin and, consequently, the direction of plant growth and morphogenesis.

## 4. Discussion

Studies have shown that the shading response is mainly caused by a combination of reduced light intensity and increased levels of FR. Currently, research on the effects of FR on plants focuses on its effects on plant morphology and growth. However, a thorough investigation into how FR specifically affects the growth of tobacco leaves has not been undertaken. To elucidate the mechanism of FR on tobacco leaf growth, this study selected different tobacco varieties and set up different far-red light environments to clarify the physiological mechanism of far-red light on tobacco leaf growth, elucidate the mode of FR on tobacco leaf growth, and identify the key signaling pathways. This provided a basis for explaining the mechanism of the shade response and improving the tobacco planting density.

### 4.1. Leaf Area of Tobacco Seedlings Under Far-Red Light Was Reduced, and Thus, Inhibited Leaf Growth

In poorly lit vegetative environments with high FR, plants utilize the shade response as a crucial survival strategy to adapt and flourish. This response involves a notable decrease in leaf size, which is achieved by actively restraining the cell proliferation and/or expansion. Leaf size decrease is a distinctive feature in the shade response, enabling plants to effectively acclimate to their light-deprived surroundings and support their growth and development. Two different cellular strategies demonstrate that the shade response limits the leaf size [29]. The shade-induced restriction of cell division during early vegetative growth and the restriction of cell expansion during late vegetative growth are mainly due to the regulation of a number of major signaling channels by plant photoreceptors [20]. Far-red light had a significant effect on the size and morphological characteristics of tobacco plant leaves compared with white light and other light treatments. Our study showed that exposure to far-red light (T3 and T4) also resulted in smaller but thinner leaves, as the plants prioritized vertical growth over expanding their leaf surface area. This affected the overall photosynthetic efficiency of the plant, as smaller leaves are essential for capturing light and carrying out photosynthesis. This effect is part of the shade avoidance syndrome, where a plant reduces its leaf area to allow it to effectively adapt to light-poor environments and support its growth and development. Although the tobacco leaves in the T1 treatment group became larger, the leaves of tobacco grown under T2, where white light was also present, were significantly reduced. This was due to the lower R/FR ratio under T2 (R/FR = 8) and the highest R/FR ratio under T1 (R/FR = 24). The far-red light ratio under T2 had exceeded a threshold, which put it in the same “shade” environment. Similarly, although far-red light (T3 and T4) mostly resulted in a reduced leaf area, there were differences between T3 and T4. This was also caused by the higher R/FR ratio under T4 (R/FR = 1) compared with T3 (R/FR = 0.5). The T3 group was exposed to more far-red light, and its leaf area was also less. An additional point should be noted: the red-light peak of our experimental light source was observed at ≈625 nm, slightly below the canonical 660 nm value reported for PhyB in the literature. This deviation is not an experimental error but is attributable to measurement conditions. Figure 1 presents absorption spectra recorded from intact leaves with a portable spectrometer; leaf thickness, background chlorophyll absorption, and light scattering collectively induce a systematic blue-shift of ~10–15 nm, a phenomenon well documented in spectra of living tissues [35]. Importantly, the 640 nm red light still lies fully within PhyB’s effective absorption band (630–670 nm); hence, this shift has no substantive impact on our conclusions.

In the far-red light treatments (T3 and T4), we found a significant reduction in the fresh dry mass of leaves compared with the plants that underwent other light quality treatments, probably due to a shift in the allocation of resources to the stem and the height of the plant. We also found differences in the leaf development in two different tobacco varieties. For example, the leaf size of *Xiangyan No.7* was increased under T1, while the leaf size of *Yunyan87* was decreased under T1 (Figure 2b). This may have been due to the difference in the light requirement between the two tobaccos. *Xiangyan No.7* has a strong shade tolerance as well as a wider range of adaptation to light intensity, and can grow better even under weaker light conditions. In contrast, *Yunyan87* is less shade-tolerant and could not grow normally under weaker light conditions. In addition, we found that the difference in fresh dry mass was greater in *Xiangyan No.7* than in *Yunyan87* under T2, which indicates that the moisture content of *Xiangyan No.7* was higher under this light quality treatment. This may have been because T2 (WL+R) had different effects on the water uptake and retention capacity of different tobacco varieties, and the same phenomenon was observed under T4 (WL+R+FR), probably for the same reason.

### 4.2. Significant Changes in Photosynthetic Characteristics of Tobacco Seedlings Under Far-Red Light

Decreased R/FR ratios resulted in reduced chlorophyll levels in maize leaves across various sampling periods under different N application conditions [36]. The changes in chlorophyll concentration are significant because chlorophyll is critical for absorbing, transmitting, and converting light energy. In addition, chlorophyll levels and components have a major impact on the light capacity of leaves, which is directly affected by their photosynthetic capacity. It was found that tomato plants irradiated with lower R/FR spectral irradiation showed a decrease in leaf area and chlorophyll levels, whereas their leaf thickness and fruit biomass increased. In addition, this type of spectral irradiation accelerated the flowering and fruiting of the tomato [37]. However, it was observed in soybean that shading or low-light conditions increased the accumulation of cyst-like granules and photosynthetic pigment content, and led to a decrease in the net photosynthesis rate [38]. The contents of photosynthetically active pigments in chrysanthemum leaves show a similar phenomenon [39]. Our study found that exposing tobacco leaves to far-red light (T3 and T4) significantly increased the chlorophyll a, chlorophyll b, and total chlorophyll contents, whereas the chlorophyll a, chlorophyll b, and total chlorophyll contents were reduced under red light (T1 and T2). This response to red versus far-red light is mainly driven by the following physiological mechanisms: (1) Plants perceive the ratio of red-to-far-red light through photosensitive pigments. Under the canopy or in the shade, red light is heavily absorbed by the leaves above, while far-red light penetrates more efficiently, resulting in a lower ratio of red-to-far-red light. Photopigments detect this change in light quality and shift from the active Pfr form to the inactive Pr form. This conformational change can promote the gene expression of chlorophyll synthase, thereby increasing chlorophyll synthesis. (2) Far-red light exposure also leads to the upregulation of genes essential for chlorophyll synthesis. This process is mediated through a photosensitive pigment system that affects transcription factors that promote or repress these genes. Increased chlorophyll content not only improves the plant’s photosynthetic capacity but also serves as a stress tolerance adaptation. These physiological responses are integrated into broader adaptive strategies of plants to optimize survival and growth under different light conditions. Chlorophylls a and b are two key photosynthetic pigments. Chlorophyll a acts as the primary photosynthetic pigment and is responsible for capturing light energy and transferring it to P680 in photosystem II (PSII) and P700 in photosystem I (PSI) to produce high-energy electrons [40]. Chlorophyll b, on the other hand, acts as a secondary pigment, which assists in the transfer of captured light energy to chlorophyll a to enhance the efficiency of photosynthesis [41,42]. The ratio of chlorophyll a to chlorophyll b influences chloroplast photosynthesis, with elevated FR levels commonly reducing this ratio in most plant species [38,43,44,45]. Reducing the Chl a/Chl b ratio increases the reducing capacity of 2,6-dichlorophenol indophenol, thus improving the photophosphorylation activity of chloroplasts [46]. Chlorophyll fluorescence results from changes in the energy levels of the chromophore moiety. Chlorophyll fluorescence is a key indicator of photosynthesis, reflecting the absorption, transfer, and loss of solar energy by plants [47,48]. An increased NPQ is an important photoprotective mechanism for plants under adverse conditions. Through the action of the lutein cycle and PsbS proteins, plants can dissipate excess light energy in the form of heat, thereby protecting photosystem II (PSII) from photodamage. Although an increased NPQ helps protect photosystems, this protective mechanism comes at the expense of photosynthetic efficiency. Under adverse conditions, plants dissipate excess light energy by increasing the NPQ, leading to a decrease in photochemical efficiency (PSII) and electron transfer rate (ETR), which ultimately reduces the photosynthetic efficiency [49]. This could be the reason why the NPQ of tobacco leaves was significantly reduced by the addition of far-red light under the unfavorable conditions of low light intensity (105 μmol m^−2^s^−1^).

### 4.3. Anatomical Analysis of Tobacco Seedlings Under Far-Red Light and Validation of Related Pathways

**Figure 10:**
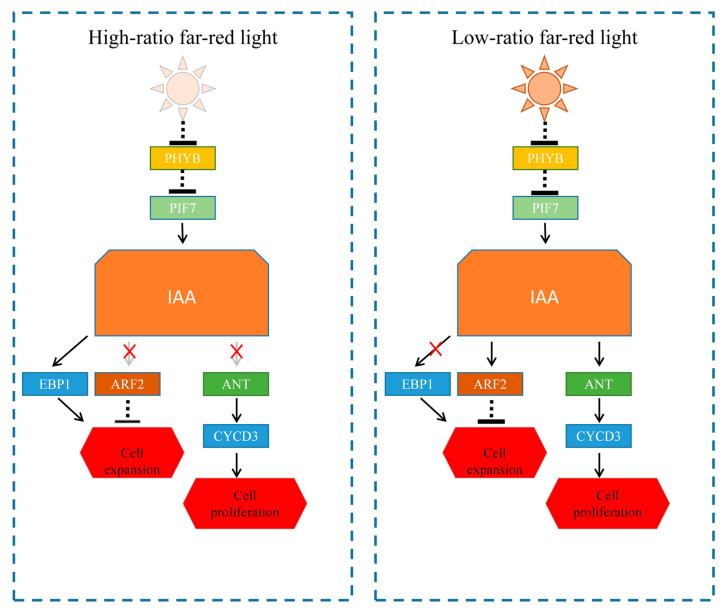
Predicted mechanisms of cell proliferation and expansion with different FR ratios. ↓ is positive regulation, 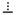 is negative regulation.

The leaves are the major location for photosynthesis in higher plants. Inside the leaf, the chloroplastic tissue regulates the light transport of photosynthesis. Plant leaves can change the structure of the chloroplastic tissue to adapt to different environments [50]. Many studies have shown that increasing FR increases the net photosynthetic rate but decreases the leaf thickness [7,11,19]. The possible reason for this may be that, under low-R/FR conditions, the cell size and cell number of the mesophyll tissue are reduced, thus lowering the leaf blade thickness [16,51]. In our study, we found that the spongy and fenestrated tissue thicknesses of tobacco leaves were significantly reduced under FR. However, the epidermal cell size of the tobacco leaves under FR was significantly increased, which seems to be different from the above studies. Therefore, to explain this phenomenon, we verified the genes and pathways involved in the cell proliferation and growth of tobacco leaves. Under shading conditions, a low R/FR ratio causes the photosensitive pigment *PHYB* to shift to an inactive state. As the amount of the inactive form of *PHYB* increases, its activity decreases, which decreases the repressive effect on the *PIFs* transcription factors. *PIFs* are activated and can promote the expression of growth-related genes, which enhances plant growth. It has been shown that PIF4 and *PIF7* are major proteins that regulate thermomorphogenesis and the shade response, respectively [28,52]. At the same time, the addition of FR leads to the progressive inactivation of Arabidopsis *PHYB*, which increases the growth-promoting effect of the *PIF7* transcription factor [53,54]. *PIF7* regulates tobacco leaf growth through the typical pif–auxin (IAA) pathway. The IAA content in tobacco leaves was increased under FR (Figure 9). Auxin is one of the key hormones for plant growth and development and has an important effect on leaf growth and development. Auxin plays a central role in the morphogenesis of plant leaves. It regulates the unfolding of leaf primordia and affects the degree of leaf flattening, which, in turn, affects the efficiency of photosynthesis [55]. Auxin affects leaf cell division, elongation, and differentiation through its signaling pathways. For example, members of the *ARF* family regulate the expression of Auxin-responsive genes in leaves [55,56]. Auxin inhibits the expression of the *ARF2* gene, which encodes an auxin-responsive transcription factor that regulates the organ size by inhibiting cell expansion [57]. The Auxin-induced expression of *ARF2* in tobacco leaves under FR decreased, which, in turn, promoted cell expansion. The *ANT* gene was induced by Auxin and is involved in regulating organ size by modulating the cell number [58,59], but the activity of *ANT* was decreased under FR. Furthermore, its downstream response gene *CYCD3* was also inhibited by FR. Auxin signaling could regulate organ size by modulating the stabilization of *EBP1* associated with the expansion of cells [22]. *EBP1* in tobacco leaves under FR was induced by Auxin, which, in turn, promoted cell expansion (Figure 10). In conclusion, low R/FR ratios converted *PHYB* to an inactive form, and *PHYB* inactivation reduced the repression of the transcription factor *PIF7*, which facilitated auxin synthesis through the typical pif–auxin (IAA) pathway. However, a low R/FR limited the proliferation of chloroplasts in the tobacco leaves by inhibiting the interaction of auxin on *ANT* and its downstream gene *CYCD3*, which, in turn, suppressed the leaf area and biomass. However, a low R/FR enhanced the expansion of epidermal cells in the tobacco leaves by promoting *EBP1* and inhibiting *ARF2*, which may have been responsible for the changes in leaf shape. Our study demonstrated that far-red light restricted the growth of tobacco seedling leaves by inhibiting the cell number, but not the cell size, of the tobacco leaves. Meanwhile, the pathways by which far-red light affected tobacco leaf growth were predicted. This provides a theoretical basis for the effect of FR on tobacco leaf growth and the shading response.

## 5. Conclusions

Our study demonstrated for the first time that FR reduces the leaf size and alters the leaf shape by inhibiting cell proliferation rather than cell expansion in tobacco leaves. We verified that FR affects the relevant genes *(NtPHYB*, *NtPIF7, NtCYCD3*, *NtEBP1*, *NtARF2*, and *NtANT*) in tobacco growth signaling and preliminarily demonstrated that FR induced tobacco growth through the *NtPHYB–NtPIF7–IAA* signaling pathway. This finding provides a new perspective to elucidate how far-red light affects plant leaf growth and its response to shade conditions. Meanwhile, we propose the signaling pathway by which FR leads to changes in leaf growth, and we propose a model for the effect of FR on the growth and shade responsiveness of tobacco leaves. Understanding these light quality effects is critical for managing light environments in agriculture, especially in controlled environments, such as greenhouses, where the spectrum can be manipulated to influence crop growth and productivity.

## Data Availability

All data supporting the findings of this study are available within the paper and its Supplementary Information.

## Supplementary Materials

The following supporting information can be downloaded at https://www.mdpi.com/article/10.3390/plants14162520/s1, Figure S1: Changes in tobacco Plabs under different light treatments; Figure S2: Arachnograms of chlorophyll fluorescence parameters; Table S1: Tobacco seedling planting environment setting parameters; Table S2: Information and primers for tobacco homologues involved in leaf development and growth.

## Abbreviations

SAS	Blindfold syndrome.
OJIP curve	Fast chlorophyll fluorescence induction curve.
FV/FM	Maximum fluorescence ratio.
NPQ	Non-photochemical quenching of chlorophyll fluorescence.
QP	Quantum yield of chlorophyll fluorescence.
PSII	Optical system II.
