# Supplementary material for: The Effect of Far-Red Light on the Growth of Tobacco Leaves
Source: Plants (Basel). 2025 Aug 13;14(16):2520. doi: 10.3390/plants14162520 (PMC12389629; doi:10.3390/plants14162520)
Supplement: Supplementary file 1 [file plants-14-02520-s001.zip › plants-3770591-supplementary.pdf]

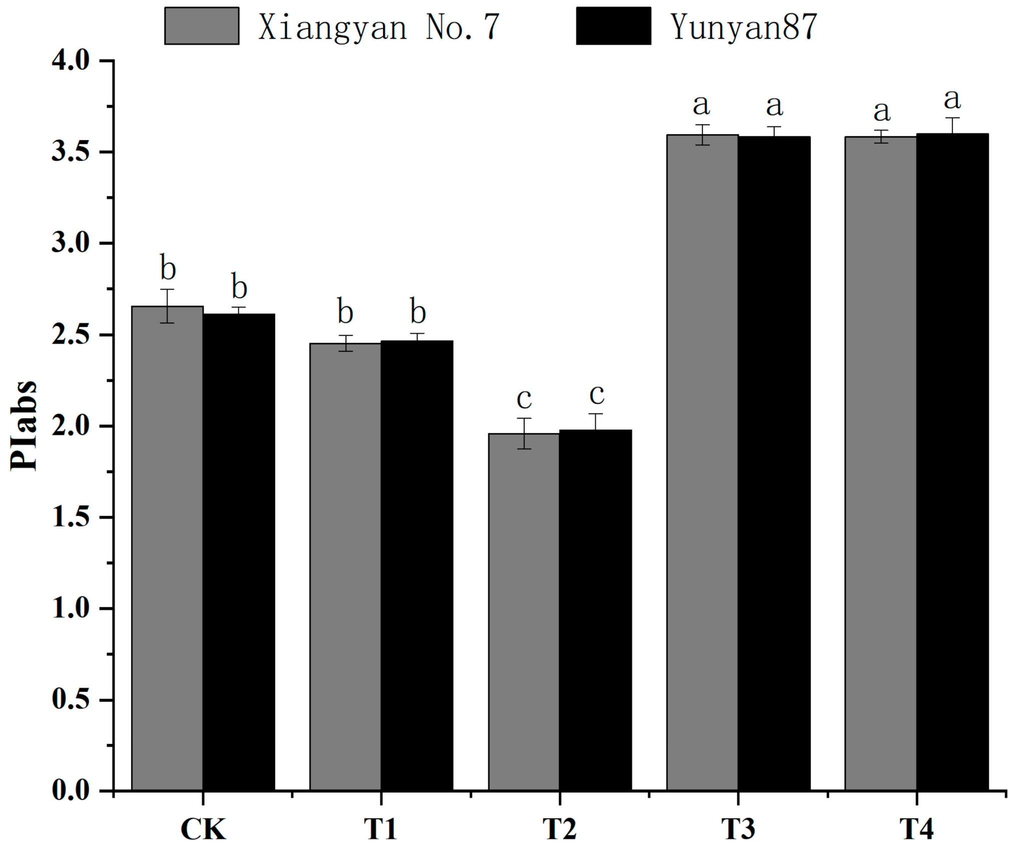

Figure S1. Changes in tobacco Plabs under different light treatments. Different letters indicate that the data are significantly different from each other (P < 0.05).

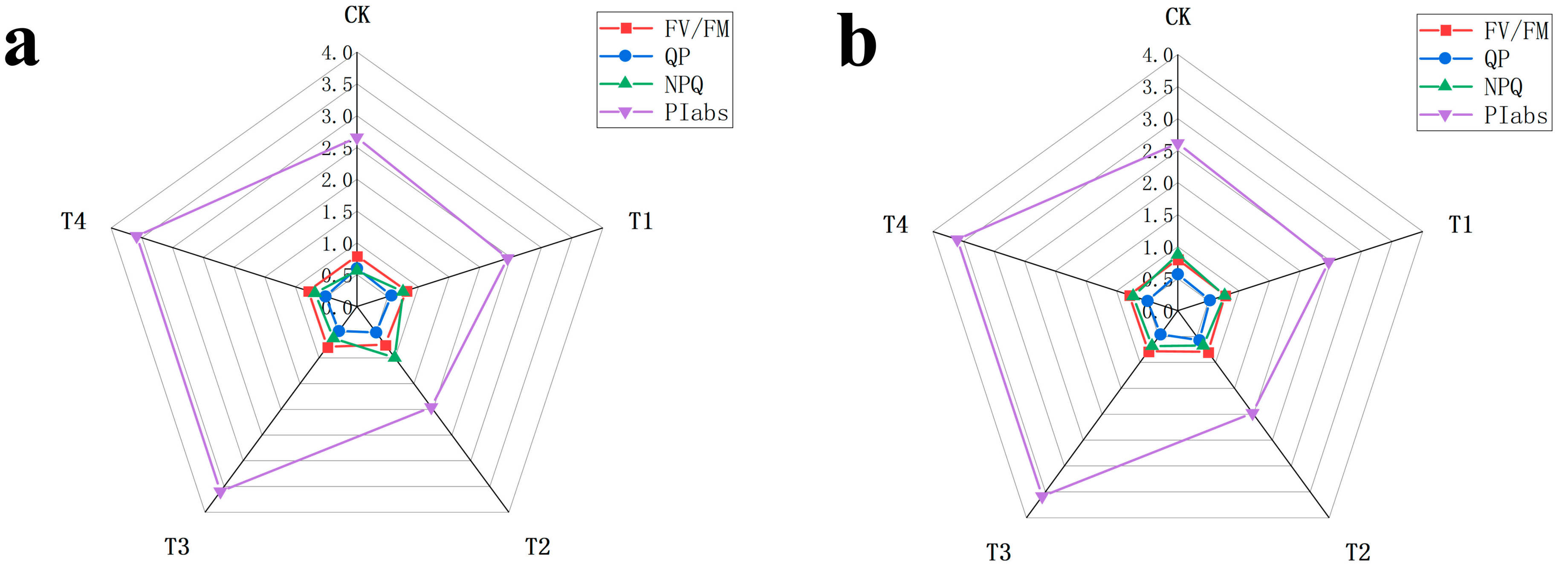

Figure S2. Arachnograms of chlorophyll fluorescence parameters. a is a spider web plot of the chlorophyll fluorescence parameters of Xiangyan No.7 under different light quality conditions; b is a spider web plot of the chlorophyll fluorescence parameters of Yunyan87 under different light quality conditions.

Table S1. Tobacco seedling planting environment setting parameters

Group	Light quality (R:FR)	Light intensity	Photoperiod	Temperature	Humidity
CK	White light (12)	105± $20\mu mol\cdot m^{-2}\cdot s^{-1}$	16h	24±2℃	70±10%
T1	Red light (24)				
T2	White + red light (8)				
T3	White + far red light (0.5)				
T4	White + red + far red light (1)				

Table S2. Information and primers for tobacco homologues involved in leaf development and growth

Function	Gene	Gene ID	qPCR-Primers
Photoreceptor	*NtPHYB*	ALN38804.1	TTATCCGACACCGACAAGAAGA<br>TGGCGGAAGGTACATCGTAAA
Phytochrome Interacting Factor	*NtPIF7*	LOC107832074	GCATCAATGTTGGACGAAG<br>AAGGCATCTGAATATGTGGTAG
Cell proliferation	*NtCYCD3*	LOC107832697	AAGTTGAGCCCTGTAATGC<br>GGTGAATAGGTGGAAGAAG
	NtANT	LOC107809390	GGTGGACAGGTAGGTATGA<br>AACGGAAAGTTAATGTGAGT
Cell enlargement	*NtARF2*	LOC109223515	CTGGTCAAGGTGGTGATAC<br>TTTCCCTCTTTCGGTTTCA
	NtEBP1	LOC109210191	CAGCCCTATCCTGTTCTAC<br>TTGTGATCCTATCTGACCC
	NtActin		GGTCGTACCACCGGTATTGTG<br>GTCAAGACGGAGAATGGCATG
